# Diversity and ecological potentials of viruses inhabiting in the Kermadec and Diamantina trench sediments

**DOI:** 10.1093/ismeco/ycaf147

**Published:** 2025-08-28

**Authors:** Pudi Wang, Xiaotong Peng, Hongmei Jing

**Affiliations:** State Key Laboratory of Deep-Sea Science and Intelligent Technology, Institute of Deep-sea Science and Engineering, Chinese Academy of Sciences, 28 Luhuitou Road, Sanya 572000, China; University of Chinese Academy of Sciences, Beijing 100049, China; HKUST-CAS Sanya Joint Laboratory of Marine Science Research, Chinese Academy of Sciences, Sanya 572000, China; State Key Laboratory of Deep-Sea Science and Intelligent Technology, Institute of Deep-sea Science and Engineering, Chinese Academy of Sciences, 28 Luhuitou Road, Sanya 572000, China; State Key Laboratory of Deep-Sea Science and Intelligent Technology, Institute of Deep-sea Science and Engineering, Chinese Academy of Sciences, 28 Luhuitou Road, Sanya 572000, China; HKUST-CAS Sanya Joint Laboratory of Marine Science Research, Chinese Academy of Sciences, Sanya 572000, China

**Keywords:** Hadal trench, viral community, ecological functions, driving force, virus–host interaction, genomic metrics

## Abstract

Viruses are the most abundant biological entities in marine ecosystems, playing an important role in biogeochemical cycling and the regulation of microbial dynamics. However, their assembly driving force, genomic evolution, and potential ecological functions in the hadal trench remain largely unknown. Here, 32 359 viral operational taxonomic units were derived from metagenomes of 40 sediment samples in the Kermadec and Diamantina trenches. High novelty and habitat-specific endemism of viruses based on the protein-sharing network analysis were demonstrated. Their auxiliary metabolic genes were involved in the biogeochemical cycles and compensatory metabolic process of the host inferring from the virus–host linkage prediction. Distinct viral community assembly in the two trenches and among different sampling depths was mainly driven by the stochastic processes, especially dispersal limitation. This was further proved by the low genomic mutation rates at deeper depths with potentially high hydrostatic pressures. These niche-dependent distribution patterns and genomic features together reflected the survival and adaptative strategy of viruses. This study provided new insights into the high diversity, ecological potentials, evolution, and adaptive mechanism of viruses in the deep biosphere.

## Introduction

Deep sea is characterized by low temperatures, high pressures, low oxygen, absence of light, and oligotrophy [1]. Despite the extreme environmental characteristics, it holds 75% of the global ocean’s prokaryotic biomass and more than 50% of the prokaryotic productivity [2]. By far, most studies on deep-sea microbe focus on the diversity, function, and ecology of prokaryotes [3]. Distinct prokaryotic community structure between epipelagic and abyssopelagic waters existed, and varied with different sediment depths [4, 5]. Meanwhile, deep-sea microbes play key roles in biogeochemical cycles of carbon, nitrogen, and sulfur [6], and perform important functions in maintaining the diversity and function of deep biosphere.

Viruses, as important component of ecosystems, are the most abundant biological entities in marine ecosystem [7]. Viruses have been shown to lyse the host, releasing intracellular organic matter from the host, influencing the material cycle and energy flow of the ecosystem [8], increasing the genetic diversity of host through horizontal gene transfer and regulating microbial community structure [9]. Viruses can also regulate host metabolism through the expression of auxiliary metabolic genes that influence microbial metabolic activity and aid in microbial-driven biogeochemical cycling processes [10]. In deep-sea sediments, viruses exhibited high lytic activity that increased with depth [11], and contained numerous auxiliary metabolic genes, enhancing the ecological functions and adaptability of their hosts [12]. Different deep-sea habitats supported distinct viral communities and harbored a wide array of novel species [13]. Therefore, in-depth research is needed to explore the composition, diversity, functions, and metabolic mechanisms of viruses in typical deep-sea habitats.

Hadal trenches are steep, narrow depressions in the ocean, and their V-shaped structure could accumulate diverse of organic sources to sustain the microbial ecosystems [14]. Viruses are widespread in the waters and sediments of Mariana trenches, including a large number of novel species [15]. They contain a rich and novel array of auxiliary metabolic genes related to carbon and nitrogen cycling, which may be associated with the ecological function and deep-sea adaptation of their hosts [16]. Viruses can infect key chemoautotrophs, subsequently influencing the composition and function of trench ecosystems. In addition, viral communities exhibit varying degrees of spatial exchange, however, by far most of the current viral studies were limited to a single trench, and comparative studies among different trenches were still very rare [17].

Kermadec trench is one of the deepest trenches on Earth, located in the southern Pacific Ocean, and has a maximum depth of 10 047 m [18]. Diamantina trench is located in the southeastern Indian Ocean, with a maximum depth of ~8047 m [19]. The two trenches differ not only in depth but also in their tectonic and geological settings. The Kermadec trench represents a typical tectonic convergent boundary, whereas the Diamantina trench is a fracture zone. Their distinct orientations and locations across different ocean basins may result in unique biogeochemical and microbial characteristics. Composition, distribution, and function of prokaryotes in the two trenches had been briefly studied [20, 21]. Based on surface sediments of several sampling sites in the Kermadec trench, high levels of novel viruses had been revealed [17]. Although both trenches are located in the Southern Hemisphere near Australia, considering their varied geological structures and hydrochemical characteristics might influence the composition and function of microbial ecosystems in different ways, it will be necessary to conduct comparative studies with inter and intra trenches. This study aimed to uncover (i) the diversity and distribution of trench viral communities, (ii) the viruses lifestyle and host-interactions, and (iii) auxiliary metabolic genes of trench viruses and their potential biogeochemical functions in the extreme deep biosphere.

## Materials and methods

### Sample collection

**Figure 1:**
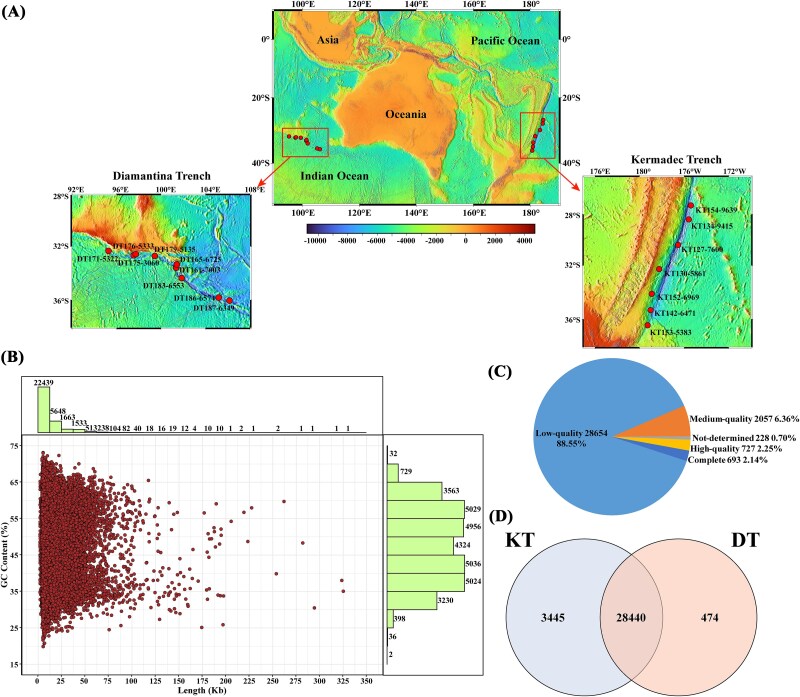
Overview of viruses in the trench sediments. (A) Geographic distribution of sampling sites. (B) Distribution of vOTUs by length and GC content. The bar charts indicate the numbers of vOTUs in each category. (C) Distribution of vOTUs assessed for quality using CheckV. (D) Venn diagram of different trenches.

Sediment samples were collected from the Kermadec trench and the Diamantina trench using a push core during cruise TS29 on the R/V “Tan Suo Yi Hao” from Nov. 2022 to Feb. 2023 (Fig. 1A and Supplementary Data 1). *In situ* hydrographic parameters (including location, depth, temperature, and salinity) were recorded by the manned submersible FENDOUZHE. All intact push core samples were immediately subsampled into 2-cm layers and stored at −80°C for further analysis. Each sediment layer was labeled as“xL”, where“1 L”represent the 0–2 cm layer,“2 L” represent the 2–4 cm layer, and so on.

### DNA extraction, sequencing, and metagenomic assembly

Total DNA was extracted from sediment samples (~0.5 g per sample) using the PowerSoil DNA Isolation Kit (MO BIO Laboratories, Inc., Carlsbad, USA), following the manufacturer’s protocol. The concentrations of extracted DNA were measured with a Qubit® 2.0 fluorometer (Life Technologies, USA). Library preparation and sequencing were carried out using the Illumina platform (Illumina, USA). Raw sequencing data were quality-filtered using fastp v0.23.4 [22] by removing adapters, low-quality reads, and reads containing poly-N sequences, to obtain clean reads (150-bp paired-end). Quality-controlled reads from each metagenome were then individually assembled using MEGAHIT v1.1.3 [23] (--k-min 21 --k-max 141 --k-step 10).

### Generation and analysis of prokaryotic metagenome-assembled genomes

The contigs assembled from the metagenome were binned by the MetaWRAP v1.3.2 [24] binning module based on metabat2, maxbin2, and concoct methods. The original bins were refined using MetaWRAP v1.3.2 [24] bin_refinement module (−c 50 -m 10), which were then quality checked by CheckM v1.2.2 [25]. The high- and medium-quality bins (completeness ≥50% and contamination ≤10%) were then aggregated and dereplicated at 95% ANI using dRrep v3.5.0 [26]. Metagenome-assembled genomes (MAGs) were taxonomically assigned using GTDB-tk v2.4.0 [27] based on classify_wf workflows. The concatenation of 120 bacterial or 53 archaeal marker genes produced by GTDB-tk were aligned and trimmed using MAFFT v7.525 [28] and trimAl v1.4.rev15 [29] with default options, respectively. Maximum-likelihood phylogeny of MAGs was inferred using IQ-TREE v2.3.4 [30] (−m MFP -alrt 1000 -bb 2000) and visualized using iTOL v6 (https://itol.embl.de/). RPKM values were used to represent relative abundances of prokaryotic MAGs, with clean reads mapped to MAGs using CoverM v0.7.0 (with parameters -m rpkm --trim-min 5 --trim-max 95 --min-covered-fraction 10) (https://github.com/wwood/CoverM) to calculate.

### Viral contig identification, taxonomic assignments and life strategy prediction

To thoroughly recover putative viral contigs, three widely recognized tools were utilized with default settings: VIBRANT v1.2.1 [31], VirSorter2 v2.2.4 [32], and DeepVirFinder v1.0 [33]. Contigs longer than 2 kb were retained based on the following criteria: (i) VirSorter2 score ≥0.9 and/or the presence of at least one hallmark gene; (ii) categorization by VIBRANT as phages; (iii) DeepVirFinder score ≥0.9 and *P* < 0.05. To prevent misidentification of eukaryotic scaffolds as viral sequences by DeepVirFinder, eukaryotic sequences were identified using Contig Annotation Tool v6.0.1 [34] against the NCBI-nr database. Any viral sequence candidates that were identified as eukaryotic at phylum level were removed. The remaining scaffolds were classified as low-quality, medium-quality, high-quality or complete sequences by CheckV v1.0.1 [35] (end_to_end) with auto options. Identified viral sequences were combined and binned using vRhyme v1.1.0 [36] to generate vMAGs. To further minimize potential contamination of vMAGs, we applied two additional filtering steps following initial binning. First, we assessed the taxonomic consistency within each bin by predicting genes using Prodigal v2.6.3 [37] (−p meta -m), and assigning taxonomy with the CAT v6.0.1 [34] (−fraction 0.5) against the viral RefSeq database. Second, we identified terminase large subunit (TerL) genes using HMMScan (HMMER v3.4) [38] against the Pfam database v37.3 [39], with thresholds of E-value ≤1e-5 and bit score ≥30. Bins that contained contigs assigned to more than one viral phylum or encoded multiple TerL genes were considered mixed populations and were removed from further analysis. The remaining un-binned sequences were combined with identified viral sequences, and were regarded as vContigs. The resulting vContigs/vMAGs were dereplicated by vRhyme v1.1.0 [36] (with parameters --derep_only --method longest --derep_id 0.95 --frac 0.80) to generate virus operational taxonomic units (vOTUs). To calculate the RPKM values of each vOTU, clean reads were mapped to viral contigs or MAGs using CoverM v0.7.0 to generate coverage profiles across samples (parameters: contig mode for viral contigs, genome mode for MAGs, −m rpkm --trim-min 5 --trim-max 95).

CAT v6.0.1 [34] was applied to classify predicted genes. In short, Open Reading Frames (ORFs) were predicted from vOTU sequences by Prodigal v2.6.3 [37] (−p meta -m -c) and mapped against the NCBI viral_Refseq database (version 2024-06-18) using CAT to determine the taxonomic affiliation of vOTUs based on the Last Common Ancestor algorithm. The life strategies of vOTUs were predicted using VIBRANT v1.2.1 [31] and CheckV v1.0.1 [35] with default parameters, and further supported by functional annotation of ORFs using eggNOG-mapper v2.1.12 [40], followed by manual inspection of sequences containing lysogeny-specific genes (e.g. integrases, recombinases, transposases, excisionases, CI/Cro repressors, and parAB).

### Prediction of virus–host interactions

For better prediction of virus–host interactions, the reference genomes from GTDB-tk were used as ex-situ hosts, and all MAGs (completeness ≥50% and contamination ≤10%) obtained from all samples in this study were used as in-situ hosts. Three different methods were used to predict virus–host interactions. (i) Nucleotide sequence homology. Sequences of vOTUs and prokaryotic MAGs were compared using BLASTn [41]. Match thresholds were ≥75% coverage over the length of the viral sequence, ≥70% minimum nucleotide identity, ≥50 bit score, ≥1500 bp length, and ≤0.001 e-value. (ii) Transfer RNA (tRNA) match. Identification of tRNAs from prokaryotic MAGs and vOTUs was performed with ARAGORN v1.2.41 [42] (−t). The match criteria required that tRNA sequences identified from prokaryotic MAGs and vOTUs must exhibit 100% coverage and 100% identity in BLASTn to be considered indicative of putative virus–host interactions. (iii) CRISPR spacer match. CRISPR spacers were identified from MAGs using MinCED v0.4.2 [43] with default options. CRISPR spacers were then matched against vOTUs using BLASTn. Matches with ≤1 mismatch over the complete length, ≥95% identity and 95% coverage were considered as the putative virus–host interactions. (iv) Oligonucleotide frequency (ONF). ONF distances between vOTUs and MAGs were assessed using VirHostMatcher v1.0 [44] with default parameters, with d_2_^*^ ≤0.2 as indicative of putative virus–host interactions. When multiple hosts were predicted for a vOTU, those supported by multiple methods were selected. In case of equal support for different phyla, only predictions supported by CRISPR or tRNA matches were kept.

### Auxiliary metabolic genes identification and annotation

Viral AMGs in vOTU sequences were identified and annotated according to previously studies [13, 45] with some modifications. Briefly, the AMGs in vOTU sequences were identified by VIBRANT v1.2.1 using default parameters and using DRAM-v v1.5 [46] with a bit score ≥60 and auxiliary score (confidence of virus-encoded) ≤3. To reduce false positives from nonviral regions, AMGs predicted near phage genome ends were filtered by assessing adjacency to tRNA genes or inverted/direct repeats, and only those satisfying the defined criteria [45] were retained to avoid false positives from nonviral regions. Subsequently, the predicted AMGs were manually curated to remove invalid AMGs, such as those involved in nucleotide metabolism, DNA-related reactions, modification of viral components, ribosomal proteins, transcriptional/translational regulators, and viral invasion. Furthermore, only those AMGs flanked by or located adjacent to at least two viral-like genes were considered high-confidence viral AMGs and retained for further analysis. The relative abundance of AMGs was determined by calculating the relative abundance of vOTUs containing those AMGs in each sample. Heatmaps were generated and visualized using TBtool-II [47].

### Network analysis

Protein-sharing network analysis of vOTUs was performed by vConTACT2 v0.11.3 [48] and Viral RefSeq (v211) was used as the reference database. Briefly, all vOTUs of trench sediments in this study were compared to vOTUs from other ecosystems in previously published data: (i) Global Oceans Viromes 2 (length ≥ 10 kb, *n* = 195 728) [49], (ii) cold seeps (*n* = 2885) [50], (iii) trench sediments (*n* = 10 843) [15–17]. ORFs of all vOTUs were predicted by Prodigal v2.6.3 [37] (−p meta -m -c) and the output file of prodigal went as the input file of vConTACT2. The protein sequences of the vOTUs were grouped into protein clusters (PCs) using vConTACT2 (parameters: -rel-mode Diamond -pcs-mode MCL -vcs-mode ClusterONE). The degree of similarity between vOTUs was calculated based on the number of shared PCs. The network was visualized with Cytoscape v3.10.2 [51].

### Calculation of evolutionary metrics

The filtered reads of each sample were mapped to vOTUs (with completeness categorized as medium-quality, high-quality, and complete) using Bowtie2 v2.5.4 [52] with default parameters and genes from vOTUs were predicted using Prodigal v2.6.3 (−m -p meta). Based on bam files and predicted genes, inStrain with default parameters [53] was used to calculate population statistics and nucleotide metrics, including single nucleotide variants (SNVs/kb) and the ratio of nonsynonymous to synonymous mutations (pN/pS) at gene levels.

### Driving force of viral community

To understand the distance-decay relationship of virus communities, ordinary least squares regression was used with the “stats” package to calculate the relationship between virus communities and geographic distance/vertical depth, where pairwise geographic distances between samples were computed using the “geosphere” package.

To determine the contribution of different ecological processes to community assembly, null model analysis was carried out using the framework described by Stegen et al. [54]. The null model expectation was generated using 999 randomizations in R. Two metrics, including β-nearest taxon index (βNTI) and Bray-Curtis-based Raup-Crick (RC_Bray_), were calculated to divide community assembly into five processes, namely, homogeneous selection, variable selection, dispersal limitation, homogeneous dispersal, and drift. βNTI >2 indicates heterogeneous selection, βNTI <−2 indicates homogeneous selection. |βNTI| <2 and RC_Bray_ <−0.95 indicate homogenizing dispersal, |βNTI| <2 and RC_Bray_ >0.95 indicate dispersal limitation, |βNTI| <2 and |RC_Bray_| <0.95 indicate drift assembly [54].

### Statistical analysis

All statistical analyses were performed using R version 4.4.1. Alpha and beta diversity of viral communities based on the relative abundance of vOTUs was calculated using the “vegan” package. The Shannon and Simpson indices were tested using the Wilcoxon tests for comparison of different trenches or sampling depths. Nonmetric multidimensional scaling (NMDS) was conducted based on Bray-Curtis dissimilarities generated from vOTUs tables with viral abundance (RPKM) using the “vegdist” function. To determine the significant difference of viral community composition between different groups, a similarity analysis (ANOSIM) was performed using the “anosim” function. Spearman correlation was calculated using the “cor” function. The petal Venn diagrams were created using R code. The boxplots were created using the geom_boxplot function in the “ggplot” package. Sankey diagrams were generated and visualized using SankeyMATIC (https://www.sankeymatic.com/). Wilcoxon tests were used to compare differences in viral evolutionary metrics (pN/pS, SNVs/kb) across different trenches and sampling depths.

## Results

### Viral community of trench sediments

A total of 40 metagenomes from sediment samples in the two trenches were used to identify viral contigs by three pipelines, and ultimately 34 197 putative viral sequences (contigs ≥5 kb or ≥2 kb and circular) were recovered. All viral sequences were clustered at 95% ANI over 80% of the sequence length, generating a total of 32 359 vOTUs (Supplementary Data 2). Among these vOTUs, the GC content ranged from 19.87% to 73.13%, with length varying from 2002 to 325 179 bp, including 10 contigs length more than 200 kb (Fig. 1B). Their quality estimated using CheckV showed that 693 vOTUs (2.14%) were complete genomes, 727 vOTUs (2.25%) were high-quality, and 2057 vOTUs (6.36%) were medium-quality, with the remaining being low-quality and not-determined (Fig. 1C). Overall, the vast majority of vOTUs (87.89%) were present in both trenches, with 10.65% and 1.46% vOTUs present exclusively in Kermadec and Diamantina trenches, respectively (Fig. 1D). Only 2077 vOTUs were shared in all trench samples (Supplementary Fig. 1A). In the surface (0–4 cm) sediments, only 3763 vOTUs were shared between the two trenches (Supplementary Fig. 1B). As for the surface sediment samples (0–4 cm), more vOTUs were shared in the Diamantina trench than in the Kermadec trench (Supplementary Fig. 1C–D). Within individual sampling station, the shallow layers (0–10 cm) contained more unique vOTUs than the deeper layers (>10 cm) (Supplementary Fig. 1E–G).

**Figure 2:**
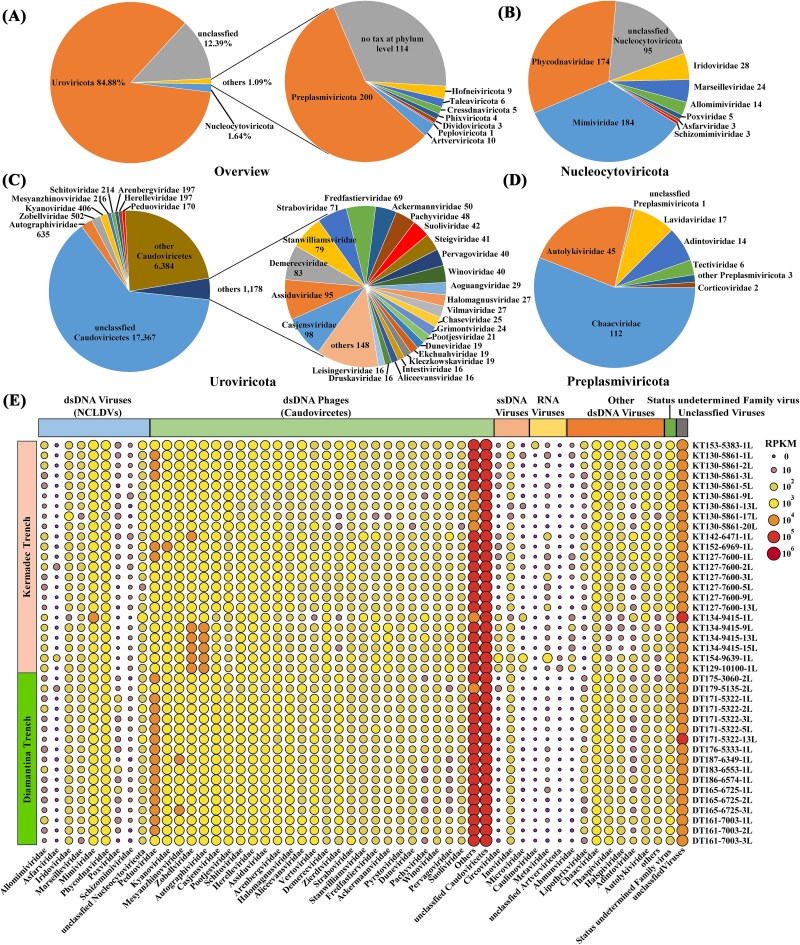
Community structure of viruses in the trench sediments. (A) Relative percentage of viral operational taxonomic units (vOTUs) at the phylum level. (B) Relative percentage of *Nucleocytoviricota*-affiliated vOTUs at the family level. The asterisk indicates vOTUs that have no taxonomic assignment at the family level. (C) Relative percentage of *Uroviricota*-affiliated vOTUs at the family level. (D) Relative percentage of *Preplasmiviricota*-affiliated vOTUs at the family level. (E) Relative abundance of viruses in each trench sediment sample at the family level.

Regarding taxonomic affiliations, 87.60% vOTUs were affiliated at the phylum level, with 84.88% assigned to *Uroviricota*, followed by *Nucleocytoviricota* (1.64%) and *Preplasmiviricota* (0.62%) (Fig. 2A). *Nucleocytoviricota*-affiliated vOTUs were grouped into eight families, with *Mimiviridae* and *Phycodnaviridae* as abundant groups (Fig. 2B). In all *Uroviricota*–affiliated vOTUs, a total of 58 families were identified, including *Autographiviridae*, *Zobellviridae*, *Kyanoviridae,* and *Mesyanzhinovviridae* (Fig. 2C). In the *Preplasmiviricota* phylum, families *Chaacviridae* and *Autolykiviridae* were more commonly observed (Fig. 2D). Regarding the community structure, in addition to a majority of unclassified viruses, a large number of dsDNA and ssDNA viruses were identified, while the abundance of RNA viruses was relatively low (Fig. 2E). At the family level, *Peduoviridae* was the predominant dsDNA virus, followed by *Kyanoviridae*. The eukaryotic NCLDVs also constituted a significant portion, with *Mimiviridae* and *Phycodnaviridae* being the most abundant groups. Distinct viral distribution between the two trenches (ANOSIM, R = 0.206, *P* = 9e-04, Supplementary Fig. 2a) and between two sampling depths (<9000 and >9000 m, ANOSIM, R = 0.384, *P =* 1e-04, Supplementary Fig. 2B) were revealed from the NMDS analysis. Shannon and Simpson diversity indices were significantly different between the two trenches (*P* < .05, Supplementary Fig. 2C) and between two sampling depths (>9000 and <9000 m, *P <* .05, Supplementary Fig. 2D).

### Novelty of trench viruses

**Figure 3:**
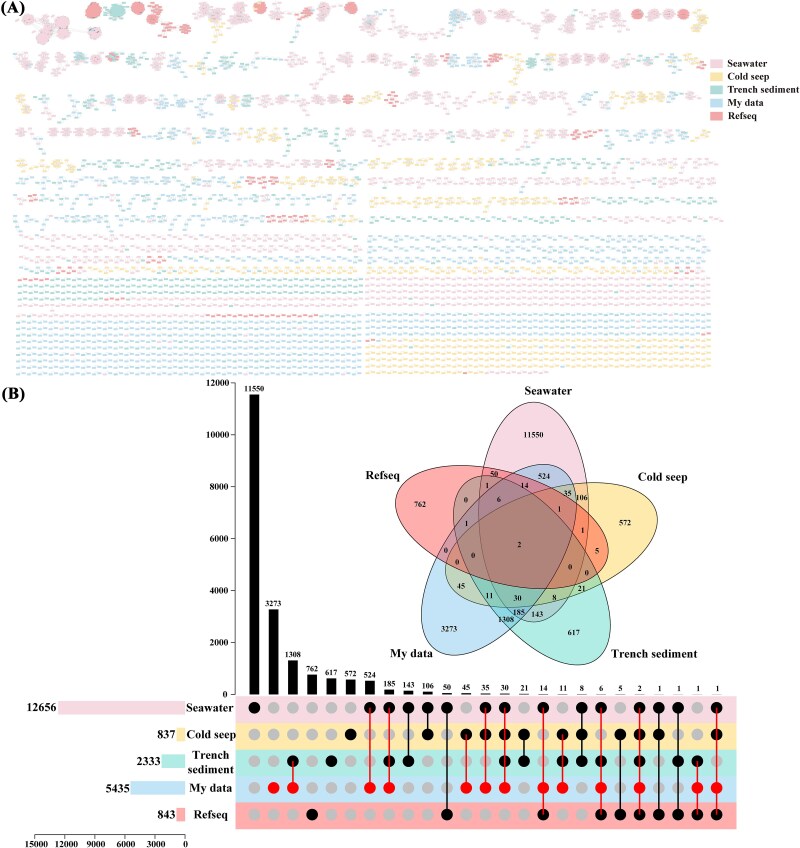
Comparative analysis of viruses from this study with RefSeq viruses and other viruses found in marine environments. (A) Gene-sharing network of viral sequences from my data with other data sets. The nodes in the network represent viruses, while the edges indicate similarity based on shared protein clusters. Node color represents the origin environment of the viruses. (B) Venn and upsets plots showing shared VCs among four environmental virus data sets and Refseq.

The protein-sharing network showed the novelty of vOTUs and their relationship to publicly available virus sequences. In total, 19 271 viral clusters (VCs) were formed, with only two VCs were shared among all marine ecosystems (Fig. 3A and Supplementary Data 3 and 4). As the most extensive marine virus database to date, the GOV 2.0 datasets contributed the highest number of VCs (*n* = 12 656), while my data, trench sediments, and cold seep databases contributed 5435, 2333, and 837 VCs, respectively. The 12 645 vOTUs recovered from this study were clustered into 5435 VCs, and had significant overlap with the trench sediments (1543 VCs), followed by the GOV 2.0 database (797 VCs) and cold seeps (124 VCs), and least with the NCBI RefSeq database (24 VC), with the remaining 3273 VCs being exclusive to trench sediments (Fig. 3B). The trench-specific VCs contained 11 487 vOTUs, with the majority affiliated as *Caudoviricetes* (*n* = 10 037, ~87.37%), followed by *Nucleocytoviricota* (*n* = 154), *Preplasmiviricota* (*n* = 119), *Dividoviricota* (*n* = 1), and ssRNA viruses (*n* = 7). Except for 31 vOTUs unassigned at the order or higher taxonomic levels, the remaining was currently unclassified.

### Viral lifestyles and virus–host linkages

**Figure 4:**
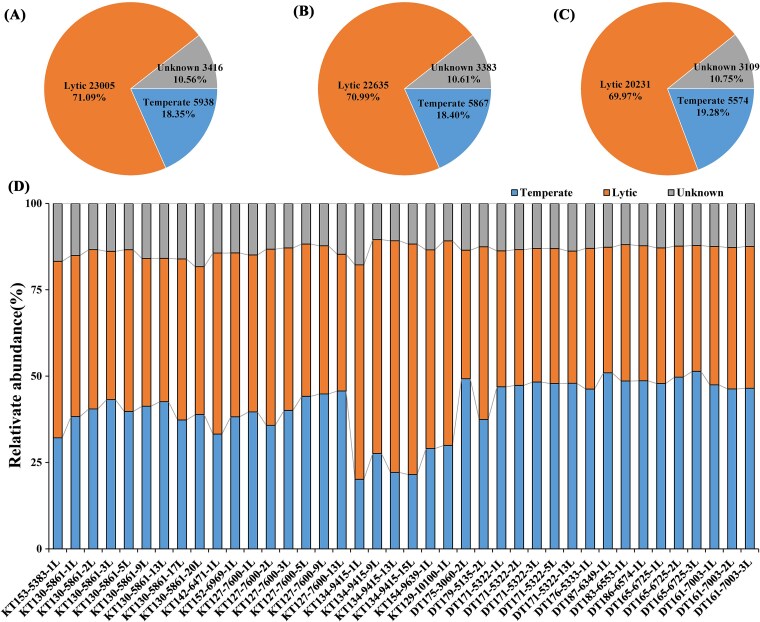
Lifestyle of viruses in the trench sediments. (A) Different lifestyle percentage of all vOTUs. (B) Different lifestyle percentage of all vOTUs in Kermadec trench. (C) Different lifestyle percentage of all vOTUs in Diamantina trench. (D) Relative abundance of vOTUs with different lifestyles across each metagenome.

Among the predicted lifestyles, 71.09% vOTUs (*n* = 23 005) were lytic, 18.35% vOTUs (*n* = 5938) were temperate, 10.56% vOTUs (*n* = 3416) were unknown (Fig. 4A). The proportions of predicted life strategies were similar in the two trenches (Fig. 4A–C). Though only 18.35% vOTUs were predicted to have a temperate lifestyle, their abundance accounted for 20.15%–51.38% in each sample, with an average of 40.87% (Fig. 4D). Temperate vOTUs accounted for a higher proportion in the Diamantina trench than in the Kermadec trench, with an average of 47.57% and 35.92% in the viral communities, respectively. Lytic viruses showed higher abundance in samples with depths exceeding 9000 m (~62.42%) compared to those from shallower depths (~42.66%).

**Figure 5:**
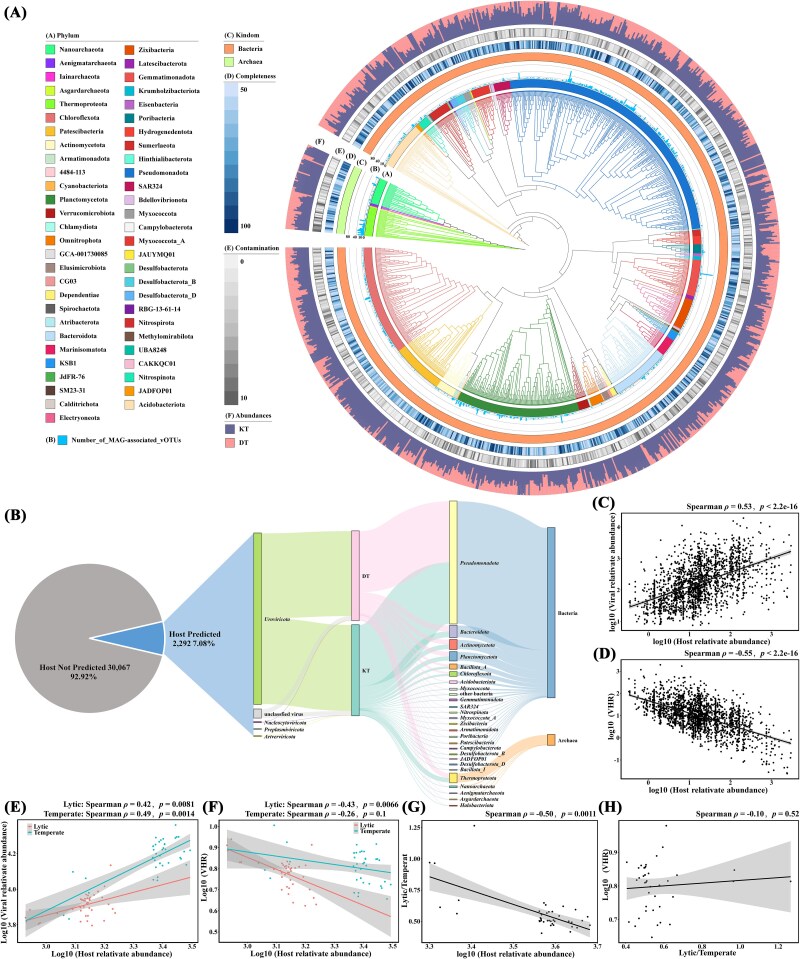
Predicted virus–host linkages in the trench sediments. (A) Maximum-likelihood phylogenetic tree of prokaryotic metagenome-assembled genomes (MAGs) inferred from the concatenated alignment of 120 bacterial or 53 archaeal single-copy marker genes. In the inter ring, clades are colored according to their annotated phylum. The next ring indicates the number of MAG-associated vOTUs. In the middle ring, MAGs from bacteria and archaea are presented in different colors. The outermost two rings show the completeness; contaminations of MAGs and the stacked columns indicate the relative abundance of MAGs at different trenches. (B) Predicted host was shown on the left; the taxonomy of predicted hosts was shown on the right. (C, D) Correlation between the relative abundance of hosts and viruses (C) or VHR (D) in trench sediments. (E, F) Associations between the relative abundance of hosts and viruses (E) or VHR (F) (temperate and lytic viruses) in different samples. (G, H) Correlation between the relative abundance ratio of lytic-to-temperate viruses and the relative abundance of hosts (G) or VHR (H).

Based on prokaryotic sequences assembly and binning, a total of 2906 high or medium-quality MAGs were dereplicated into 1100 species-level MAGs, including 1041 bacteria and 59 archaea, spanning 56 phyla (Fig. 5A and Supplementary Data 5). Within the domain Bacteria, MAGs were mainly affiliated with *Pseudomonadota* (*n* = 275), *Planctomycetota* (*n* = 131), and *Chloroflexota* (*n* = 117). Within the domain Archaea, MAGs were dominated by *Thermoproteota* (*n* = 29) and *Nanoarchaeota* (*n* = 26). Most MAGs (87%) were present in both trenches, with 12.6% and 0.4% present only in Kermadec and Diamantina trench, respectively (Fig. 5A).

Among all vOTUs, only 7.08% (*n* = 2292) had predicted hosts, resulting in 14 659 virus–host linkages (Fig. 5B and Supplementary Data 6). Those linkages were mainly predicted based on tRNA-matches (*n* = 7767), followed by nucleotide sequence homology (*n* = 4446), oligonucleotide frequency (*n* = 2443) and CRISPR-spacers matches (*n* = 128). Of these linkages, 124 were predicted by two or more methods. Among the predicted hosts, 73.05% (*n* = 11 416) were derived from the GTDB-tk database, while ~26.95% (*n* = 4213) were from this study. A total of 54 phyla of prokaryotic hosts were predicted, comprising 49 bacterial phyla and five archaeal phyla. Most vOTUs (*n* = 2271) were found to infect only a single phylum of hosts, with only a few vOTUs (*n* = 21) capable of infecting two or more phyla of host. A total of 2106 vOTUs were associated with bacteria, with the most frequently predicted hosts belonging to *Pseudomonadota* and *Bacteroidota*. Additionally, 186 vOTUs were linked to archaea, with *Thermoproteota* being the most commonly predicted host.

Positive correlation existed between the relative abundances of viruses and their hosts (Spearman ρ = 0.53, *P <* 2.2e-16, Fig. 5C), while a negative correlation was observed between the virus-to-host ratio (VHR) and host relative abundance within each trench, indicating that higher abundance hosts tend to exhibit lower VHR (Spearman ρ = −0.55, *P <* 2.2e-16, Fig. 5D). The relative abundance of temperate and lytic viruses was positively correlated with relative abundance of their hosts, but the increase in the relative abundance of temperate viruses is more pronounced (Fig. 5E). Additionally, the relative abundances of temperate and lytic viruses showed a negative correlation with VHR, with the decrease in VHR for temperate viruses being more moderate than that for lytic viruses (Fig. 5F). Based on the relative abundance, the ratio of lytic to temperate viruses were negatively correlated with host (Spearman ρ = −0.50, *P =* .0011, Fig. 5G), but positively correlated with VHR (Spearman ρ = −0.10, *P =* .52, Fig. 5H).

### Potential viral auxiliary metabolic genes associated with biogeochemical cycles

**Figure 6:**
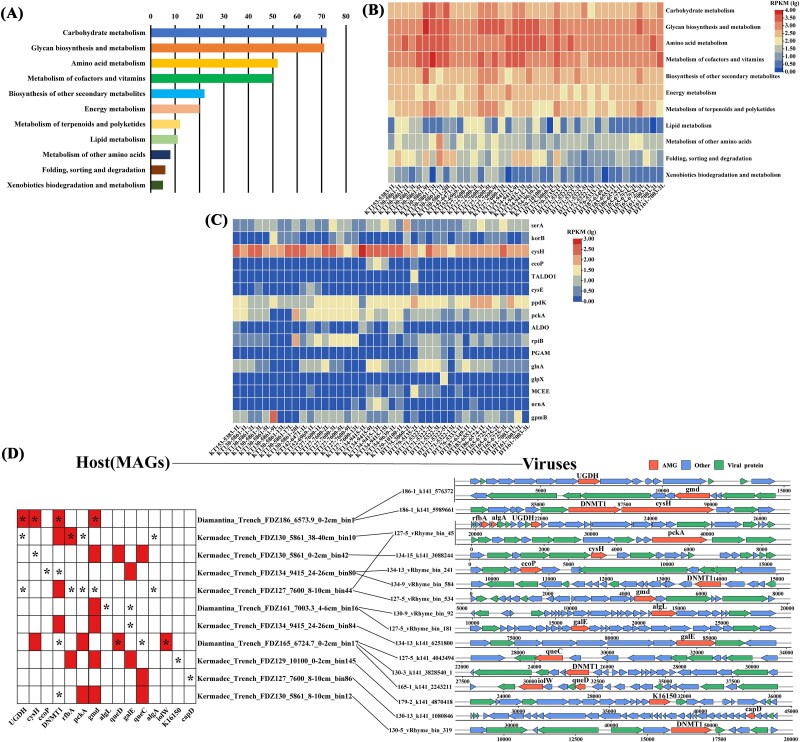
Functional classification of viral auxiliary metabolic genes (AMGs). (A) Kyoto Encyclopedia of Genes and Genomes (KEGG) annotation of viral AMGs. (B) Relative abundance (log₁₀ scale) of viral AMGs in different sediment samples. (C) Relative abundance (log₁₀ scale) of viral AMGs of energy metabolism obtained in different sediment samples. (D) Metabolic linkage of metagenome-assembled genomes (MAG) and viral AMGs. For MAG (left), the presence and absence of the homologs of viral AMGs were indicated in different colors. The asterisk indicated the AMG that was carried by the corresponding virus. For viruses (right), the genomic context of AMG was shown.

After manually curated, a total of 1930 genes recognized as putative AMGs and were categorized into 199 gene families according to Kyoto Encyclopedia of Genes and Genomes annotation (Fig. 6A and Supplementary Data 7 and 8). The majority AMGs were involved in carbohydrate metabolism, glycan biosynthesis and metabolism, amino acid metabolism, metabolism of cofactor and vitamin, biosynthesis of other secondary metabolites, and energy metabolism, in addition to the carbon (*serA, korB, TALDO1, ppdK, pckA, ALDO, rpiB, PGAM, glpX, MCEE, gpmB*), nitrogen (*glnA*), and sulfur (*CysH*, *CysK*, and *nrnA*) cycling (Fig. 6B and C). At the same station, carbohydrate metabolism generally occupied higher proportion in the deep layer (>10 cm) than the shallow layer (0–10 cm). Higher abundance of energy metabolism was detected from the Kermadec trench than the Diamantina trench (Fig. 6B and C). Meanwhile, 16 vOTUs carrying carbon and sulfur-related AMGs were predicted to infect 11 trench MAGs, resulting in 17 virus–host linkages (Fig. 6D and Supplementary Data 9). Most of the vOTUs were predicted to be temperate viruses. These vOTUs were classified as *Caudoviricetes*, with hosts spanning five different phyla. The host MAGs of 13 virus–host linkages didn’t contain homologs of the AMGs carried by the relevant vOTUs, whereas those from the other four linkages included homologs of the AMGs from the corresponding vOTUs.

### Biogeography of viral communities in the trenches

**Figure 7:**
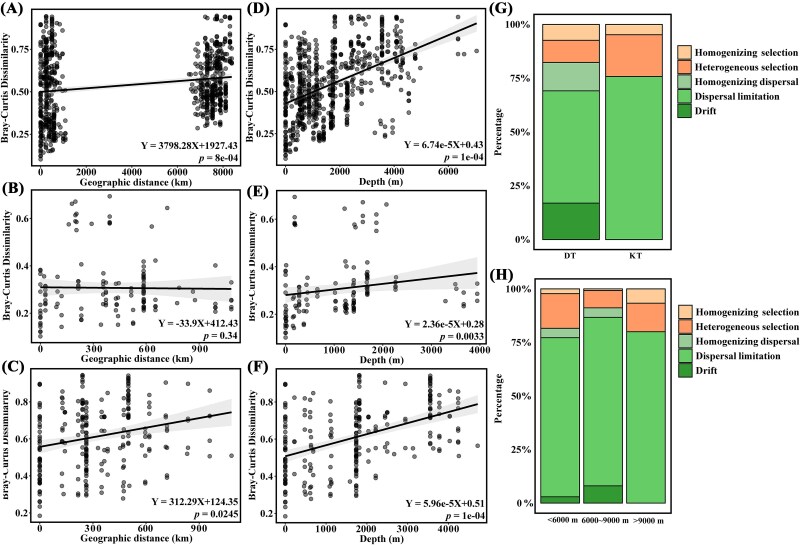
The distribution and assembly process of viral communities. (A–C) The correlation between viral community dissimilarities (Bray–Curtis distances) and geographic distances for all samples (A), Diamantina trench (B), and Kermadec trench (C), respectively. (D–F) The correlation between viral community dissimilarities and sampling depths for all samples (D), Diamantina trench (E), and Kermadec trench (F), respectively. (G, H) The relative contributions of different viral community assembly processes in the two trenches (G) and across different sampling depths (H).

Significantly negative distance-decay relationships across the geographical distance (Fig. 7A–C) and the sampling depth (Fig. 7D–F) were revealed. Along the geographic distance, significantly negative relationship was found for the integrated region (slope = 3798.28, *P =* 8e-04, Fig. 7A) and the Kermadec trench (slope = 312.99, *P =* 0.0245, Fig. 7C), but with opposite pattern in the Diamantina trench (slope = −33.9, *P =* .34, Fig. 7B). The viral community dissimilarity increased with sampling depth of the integrated region (slope = 6.74e-5, *P =* 1e-04, Fig. 7d), in the Diamantina trench (slope = 2.36e-5, *P =* .0033, Fig. 7E), and in the Kermadec trench (slope = 5.96e-5, *P =* 1e-04, Fig. 7F). The null model showed that stochastic processes, especially the process of dispersal limitation, played a more substantial role than deterministic processes in the two trenches and different sampling depths (Fig. 7F and G).

### Trench viruses are genetically conserved and under strong purifying selection

**Figure 8:**
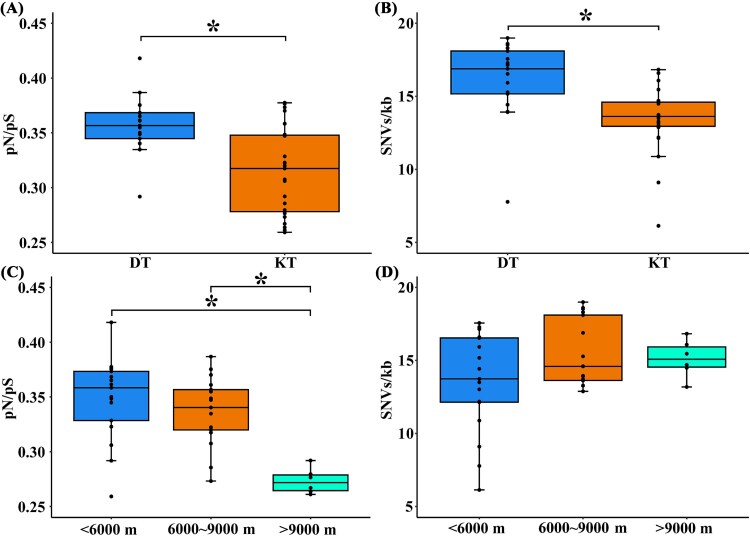
Evolutionary metrics of vOTUs in the trench sediments. (A, C) The nonsynonymous to synonymous mutation ratio (pN/pS) of viral gene from vOTUs (completeness ≥50%) in the two trenches (A) and from different sampling depths (C). (B, D) The single-nucleotide variants (SNVs/kb) of viral gene from vOTUs (completeness ≥50%) in the two trenches (B) and from different sampling depths (D).

Several evolutionary metrics, including the ratio of nonsynonymous to synonymous mutations (pN/pS) across the entire viral sequence and the frequency of single-nucleotide variations per thousand base pairs (SNVs/kb), were further explored for the medium-quality, high-quality, and complete vOTUs (completeness ≥50%). pN/pS and SNVs/kb ratios were significantly different among the different sampling depths and trenches (Fig. 8 and Supplementary Data 10). Significantly higher pN/pS and SNVs/kb were observed in the Diamantina trench than the Kermadec trench (Wilcoxon test, *P <* .05, Fig. 8A and B) In addition, samples collected from depths <9000 m exhibited significantly higher pN/pS values than those from depths >9000 m (Wilcoxon test, *P <* .05, Fig. 8C).

## Discussion

### Diversity and novelty of viruses

Three pipelines were applied to recover more viruses from metagenomes of the trench sediments. ssDNA and RNA viruses contributed only a small fraction to the total viral reads, while the high proportion of NCLDVs may be related to the binning of viral sequences using vRhyme [36, 55]. The majority of vOTUs were classified as class *Caudoviricetes*, the sole class of the phylum *Uroviricota*, which has been observed in other deep-sea sediments [13], likely due to the high proportion of *Caudoviricetes* reference sequences present in current databases.

Distinct viral distribution A pattern was shown for the two trenches, reflecting by the less shared vOTUs and significantly different viral community composition and diversity, suggested that different trenches harbored their endemic viral species [17]. Sampling depth was another key factor for the differentiation of viral communities, same as for the prokaryotic communities in trench sediments [5]. Meanwhile, only a few vOTUs from my data were clustered into VCs, most of which had no homologs in other RefSeq database, indicating the high novelty and specificity of viruses in trench sediments [13, 56]. A small number of VCs from my data were shared with GOV2, indicating less viruses in common between seawaters and sediments [49]. Only two VCs existed across all the marine habitats further proved a high degree of variation and a vast unknown diversity of viral communities in different marine ecosystems [50].

### Virus–host interactions in trenches

Viruses could significantly impact prokaryotic community dynamics, evolution, and ecology through their widespread infections and interactions with hosts [57]. In the two trenches, very few (*n* = 21) vOTUs were predicted to be capable of infecting two or more host phyla, while the majority of viruses infect only a limited range of hosts, same as found in the deep-sea cold seeps [50] and hydrothermal vents [56]. *Pseudomonadota* with classes of *Alphaproteobacteria* and *Gammaproteobacteria,* and *Thermoproteota* with class *Nitrososphaeria* were the most frequently predicted bacterial and archaeal hosts in this study. Those prokaryotic groups predominated in the Mariana trench based on metagenomes [58] and in the Kemadec and Diamantina trenches based on amplicon sequencing of 16S rRNA gene [21]. Those predominantly autotrophic prokaryotic groups were usually actively involved in the C/N/S cycles in the deep trenches [59–61], and their frequent association with viruses through infection and lysis would lead to a subsequent alteration in the microbial community structure and biochemical cycling.

Both potentially lytic and temperate viruses were found in the two trenches, with temperate viruses showing comparable relative abundance to lytic ones in many samples. Temperate vOTUs could be more widespread in the trench than currently predicted, and the underestimation was likely due to potential oversight in the annotation of lysogeny-specific genes and/or misidentified as lytic or left undetected [13]. The negative relationship between host abundance and VHR suggests that virus–host interactions may follow the Piggyback-the-Winner (PtW) model, although further validation is required. This model posited that viruses exploit hosts through temperate when host density was high, protecting host cells from infection by related viruses via superinfection exclusion mechanisms rather than killing them [62]. Generally, our data further supported that viruses in deep-sea ecosystems tend to adopt a temperate lifestyle, which could potentially promote virus-mediated horizontal gene transfer and genetic variation, and enable hosts to better adapt to the harsh deep-sea environment as well [63, 64].

### Impact of viral auxiliary metabolic genes on host metabolism and biogeochemical cycles

Viral AMGs participated in host metabolism subsequently influencing the biogeochemical processes in the deep-sea water and sediments [65]. According to KEGG annotation, the viral AMGs in this study were involved in various metabolic pathways, including carbohydrate metabolism, amino acid metabolism, cofactor and vitamin metabolism, and energy metabolism, similar to the findings in the Mariana trench [16]. AMGs were associated with carbon cycle including phosphoenolpyruvate carboxykinase (*pckA*) and fructose-bisphosphate aldolase (*ALDO*). The former catalyzes the ATP-dependent conversion of oxaloacetate to phosphoenolpyruvate, a key step in gluconeogenesis that connects the tricarboxylic acid (TCA) cycle to carbon assimilation and energy metabolism under anaerobic or heterotrophic conditions [66]. The latter participates in glycolysis and gluconeogenesis by cleaving fructose-1,6-bisphosphate into triose phosphates, and also plays an important role in the Calvin cycle and other carbon flux rearrangement pathways [67]. In addition to the glutamine synthetase (*glnA*) associated nitrogen metabolism, phosphoadenosine phosphosulfate reductase (*CysH*) and serine O-acetyltransferase (*CysE*) were major AMGs involved in the sulfur cycle. The former was involved in assimilatory sulfate reduction by converting activated sulfate to sulfite [68], whereas the latter catalyzes the biosynthesis of O-acetylserine, a precursor of cysteine, facilitating the incorporation of inorganic sulfur into organic compounds [69]. Above information suggested that trench viruses played a key role in the trench biogeochemical cycles by aiding microbes in utilizing AMGs to enhance their metabolic processes and ecological functions.

AMG characteristics were also affected by the viral lifestyle. Lytic viruses were more likely to encode AMGs that promote host reproduction, while temperate viruses tended to encode AMGs that enhance host survival [70]. In addition to the predominance of temperate lifestyle, a higher relative abundance of lytic viruses in the Kermadec trench had AMGs related to lipid metabolism and folding, sorting, and degradation, which was proposed to enhance the host’s translation efficiency, thereby promoting viral reproduction [71]. In contrast, Diamantina trench had a higher relative abundance of temperate viruses with AMGs associated with carbohydrate metabolism, energy metabolism, polysaccharide biosynthesis and metabolism, and xenobiotics biodegradation and metabolism, likely facilitating the breakdown and utilization of complex carbohydrates and enhancing the host’s environmental adaptability [72]. Based on the predicted connections between hosts and viruses carrying these AMGs, temperate virus infections may supplement the missing functions of host in the carbon, nitrogen, and sulfur cycles [73]. The absence of host genes could also due to incomplete annotations or functional redundancy rather than genuine loss. In addition, some hosts contained homologs of the AMGs carried by relevant vOTUs, suggesting that these viruses may support or complement host metabolism by encoding similar functional genes [73].

### Geographic distribution, community-driving forces, and genomic metrics

Spatial variation of viral communities in the two trenches might be attributed to the *in situ* physicochemical conditions [17], although they were both located in the south hemisphere surrounding Australia, but with different geographical features [19, 74]. Depth had been reported as a crucial factor determining the viral community structure, distribution and ecological function [15], possibly because nutrient accumulated and pressure increased with depths along the V-shaped topography of the trenches, sequentially influenced the community structure of hosts and viruses [75].

Stochastic processes were important drivers of viral assembly and biogeography in this study, although the relative importance of deterministic processes varied in different trenches. Stochastic processes had been found as the main driving forces in the construction of prokaryotic communities in the water column of the Mariana trench [76] and the sediments of the Yap trench [77]. Dispersal limitation was found as the predominant process in both trenches and different sampling depths, very likely caused by the fact that hadal trenches are geographically isolated environments, where the intrahydrodynamic forces would weaken the influence of environmental selection on species composition. Additionally, sedimentary microbes often exhibited low dispersal abilities [78]. This might explain the low variation of α-diversity among different sampling stations within the same trench. In the deterministic process, the relatively higher proportion of heterogeneity selection may cause shifts in β-diversity between the trenches and among the different sampling depths [79]. Higher proportion of homogenizing selection at depths >9000 m likely due to species facing more stable environmental conditions that drive convergence to adapt to extreme pressures.

The viral community assembly mechanism was further supported by the viral genomic features. The significantly lower pN/pS ratio and SNVs/kb in the Kermadec trench than in the Diamantina trench reflected a reduced mutation rate possibly caused by strong purifying selection pressure due to a deeper depth in the former. The lowest pN/pS ratio at depths > 9000 m indicated that the highest hydrostatic pressure acted as stronger purifying selection pressure on viruses, maintaining gene and protein stability essential for viral survival. Given the coevolutionary relationship between viruses and their microbial hosts [80], it is plausible that host adaptation to high hydrostatic pressure may influence viral genome evolution. In line with this, deep-sea viruses have been found to carry diverse AMGs that improve host metabolic performance, helping the host survive in extreme environments and thereby supporting prolonged viral infection [81]. On the other hand, the highest pN/pS ratio and SNVs/kb of viral genomes were found along the abyssal-hadal transitional zone between 6000 and 9000 m, reflecting the influence of fluctuating biotic and abiotic parameters that may drive more adaptive mutations. Overall, these observations highlight the complex evolutionary pressures that trench ecosystems impose on viruses and their hosts.

## Supplementary Material

SI_revised_ISME_commu_8_21_ycaf147

trench_8_20_supp_data_ISME_commu_ycaf147

## Data Availability

The raw metagenomic sequence data associated with this study were deposited in the National Center for Biotechnology Information (NCBI) Sequence Read Archive (SRA) under accession number PRJNA1111327. All processed data generated in this study were provided in Supplementary Data files (Supplementary Data 1–10).
