# Supplementary material for: Diversity and ecological potentials of viruses inhabiting in the Kermadec and Diamantina trench sediments
Source: ISME Commun. 2025 Aug 28;5(1):ycaf147. doi: 10.1093/ismeco/ycaf147 (PMC12448302; doi:10.1093/ismeco/ycaf147)
Supplement: SI_revised_ISME_commu_8_21_ycaf147 [file si_revised_isme_commu_8_21_ycaf147.pdf]

Supplementary materials

Diversity and ecological potentials of viruses inhabiting in the Kermadec and Diamantina trench sediments

Pudi Wang[1,2,3], Xiaotong Peng[1], Hongmei Jing[1,3*]

[1]State Key Laboratory of Deep-Sea Science and Intelligent Technology, Institute of Deep-sea Science and Engineering, Chinese Academy of Sciences;

[2]University of Chinese Academy of Sciences, Beijing, China;

[3]HKUST-CAS Sanya Joint Laboratory of Marine Science Research, Chinese Academy of Sciences, Sanya, China

Running title: Viruses in the trench sediments

* **Correspondence:** Hongmei Jing, State Key Laboratory of Deep-Sea Science and Intelligent Technology, Institute of Deep-sea Science and Engineering, Chinese Academy of Sciences, 28 Luhuitou Road, Sanya, 572000, China. E-mail: hmjing@idsse.ac.cn

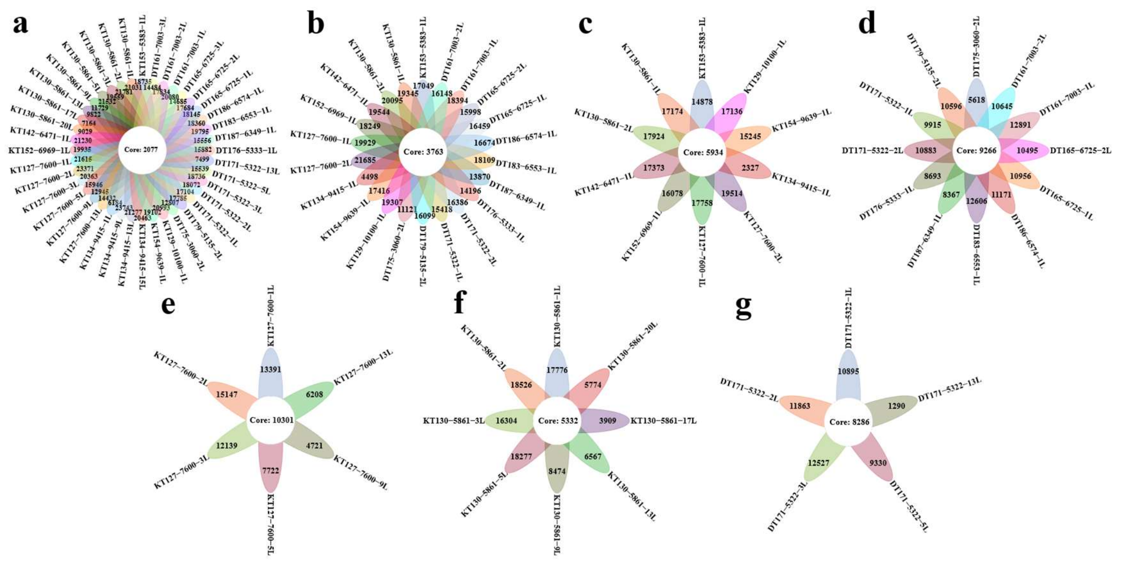

Supplementary Fig. 1 Venn diagram showing the overlap of vOTUs among different samples, including all samples **(a),** surface samples (0-4cm) in two trenches (**b**), in Kermadec trench only (**c**), in Diamantina trench only (**d**); and different layers at the Stn. KT127-7600 (**e**), Stn. KT130-5861 (**f**) and Stn. DT171-5322 (**g**), respectively. Note: KT represented the Kermadec Trench, DT represented the Diamantina trench.

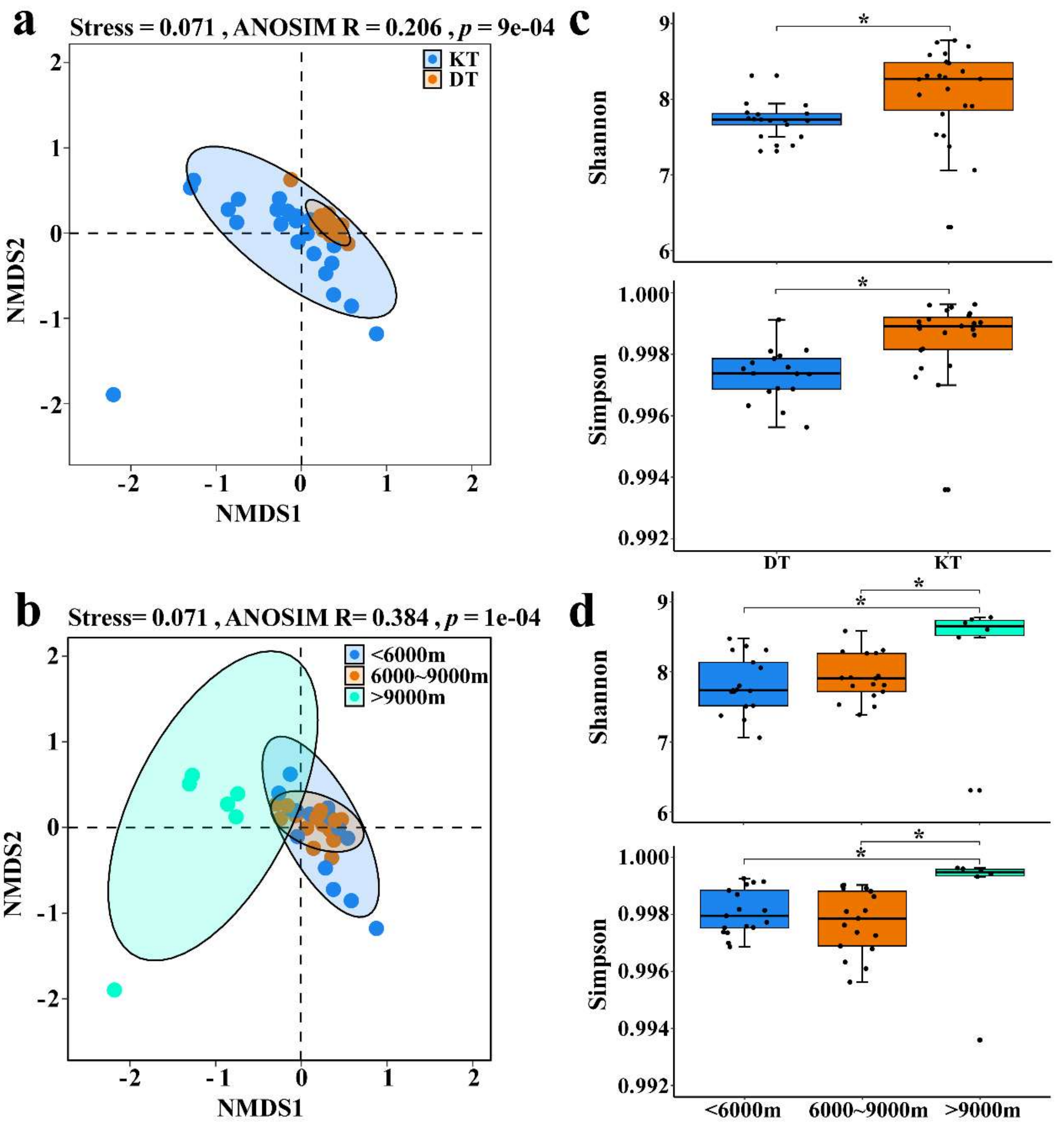

Supplementary Fig. 2 Comparison of viral communities among different samples. NMDS analysis of viral communities between two trenches (**a**) and among different sampling depths (**b**). Shannon and Simpson diversity indices of the viral communities between two trenches (**c**) and among different sampling depths (**d**).
